# Enhancing Cultural Competence in Undergraduate Nursing Students: An Integrative Literature Review of Strategies for Institutions of Higher Education

**DOI:** 10.1177/10436596241301407

**Published:** 2024-12-08

**Authors:** Khumoetsile Daphney Shopo, Vistolina Nuuyoma, Leonard Chihururu

**Affiliations:** 1 North-West University, Potchefstroom, South Africa; 2 University of Namibia, Windhoek, Namibia

**Keywords:** cultural competence, undergraduate nursing students, integrated literature review, nursing education, nursing education institutions

## Abstract

**Introduction:**

Globally, health care organizations need to provide quality, culturally congruent health care to increasingly diverse patients. Nursing education institutions must therefore produce culturally competent nursing professionals to provide safe and culturally congruent care. This review aimed to identify and outline strategies to enhance cultural competence of undergraduate nursing students.

**Methodology:**

The integrative literature review followed the five steps of Lubbe et al. searching the university Libguides, EBSCOhost, Scopus, and Google Scholar databases for literature published between 2014 and 2023 that met the inclusion criteria. The methodological quality was ensured through the John Hopkins Evidence-based Appraisal Tool.

**Results:**

Five themes emerged as strategies that can enhance cultural competence for nursing students: integrating cultural competence into undergraduate nursing curriculum, cultural immersion, innovative pedagogical approaches, role of nurse educators, and students’ assessment.

**Conclusion:**

Findings provide nurse educators with evidence-based information on strategies to enhance cultural competence of undergraduate nursing students at nursing education institutions.

## Introduction

Forming connections with culturally diverse individuals without cultural competence is a challenging endeavor (Rukadikar et al., 2022). Globally, health care organizations need to provide quality, culturally congruent health care to increasingly diverse patients; this is compounded by global movement of nursing professionals. Gradellini et al. (2021) argued that it is essential to therefore incorporate cultural competence and intercultural communication in nursing education institutions (NEIs). As a primary foundation of clinical nursing, cultural competence is crucial in enhancing patient trust, reducing health care inequalities, and improving cultural safety (Sharifi et al., 2019). Cultural competence is the vigorous process of developing the ability to deliver effective, safe, and quality care to patients by considering their different cultural aspects (Sharifi et al., 2019). Previous research indicates that nurses in culturally diverse contexts struggle with the notion of cultural competence (Almutairi et al., 2015, 2017; Shopo et al., 2023). Nursing students, through clinical practice, encounter different linguistic backgrounds, religious affiliation, ethnic or racial origins, immigrants, migrants, and refugees. Therefore, there is a need for health care professionals, including students, to be adequately trained to render culturally competent care (Zarzycka et al., 2020).

Diversity is based on a variety of factors, including beliefs, attitudes, values, languages, religion, birthplace, citizenship status, ethnicity, race, kinship and family networks, educational background, past discrimination, and bias experiences, among others (Jeffreys, 2019). Cultural competence therefore refers to how the concept of respect is practiced in ensuring that varied populations’ cultural beliefs, values, rights, and expectations are recognized in the delivery of culturally relevant health care (Rukadikar et al., 2022). Cultural competence as a strategy improves the capacity of health care systems, and providers, to render culturally responsive health care and positive patient outcomes. Cultural competence is associated with better communication during patient–nurse interactions, improved patient understanding of health issues, and compliance with recommendations for medication and lifestyle modifications (Zarzycka et al., 2020).

Cultural competence as a model of health care service delivery consists of five concepts: cultural awareness, cultural knowledge, cultural skill, cultural encounters, and cultural desire (Campinha-Bacote, 2002). These concepts of Campinha-Bacote’s (2002) model have a symbiotic relationship with each other and therefore must be addressed in every encounter with the client.

Cultural competence is a skill that can be taught, trained, and achieved (Stubbe, 2020). Nursing students from multicultural contexts experience challenges during transcultural nursing care, suggesting a lack of cultural competence (Nuuyoma et al., 2024). Owing to that, health care organizations where clinical practice is conducted, together with NEIs, have substantial roles in preparing and improving nursing students’ cultural competence. In addition, incorporating cultural competence into higher education institutions is a principal aspect of the creation of cross-cultural settings where undergraduate students and academics can expand their conceptions of how culture and belief systems influence professional decision-making (Cross et al., 2020). This is also the case in nurse–patient interactions.

Research demonstrates that nurses who share a similar culture and language as their patients score higher on perceptions of cultural competence than those who were born in different countries (Almutairi et al., 2017). However, a multi-nation study with undergraduate nursing students from Belgium, Spain, Portugal, and Turkey reports learning needs and gaps in training regarding students’ ability to provide culturally and linguistically appropriate care, as well as a lack of exposure to culturally diverse clients (Antón-Solanas, Tambo-Lizalde, et al., 2021). Moreover, findings from a recent study by Nuuyoma et al. (2024) suggest that the cultural competence of undergraduate nursing students in low-and-middle-income countries like Namibia warrants enrichment. Similarly, Mhlongo (2016) argues that cultural competency pedagogy in nursing in South Africa is inadequate, and advocates for the development of nursing education that is culturally appropriate for the low-and-middle-income country’s context.

Given this background, there is a critical need for NEIs to produce nursing professionals who are culturally competent to render health care that is culturally safe and sensitive for all patients. A gap exists in evidence-based literature, which outlines strategies that can be used to enhance the cultural competence of undergraduate nursing students at NEIs. Therefore, this review aimed to critically evaluate and synthesize recent peer-reviewed scientific literature to understand and outline strategies that can be used to enhance the cultural competence of undergraduate nursing students at NEIs.

## Method

The integrative literature review (ILR) is described as a distinctive form of research that generates new knowledge of an identified topic (Toracco, 2005). The authors followed a rigorous process guided by the five steps of ILR described by Lubbe et al. (2020).

## Composition of a Review Question

Riva et al. (2012) suggest that a strong research question can be framed using the PICOT framework. The PICOT framework refers to Population, Phenomenon of Interest, Comparison, Outcome and Timeframe. However, in line with the current ILR, the researchers in this current study applied PIOS as this framework was also applied by Nyaloko et al. (2023). The PIOS framework variation refers to P—population, I—phenomenon of interest, O—outcome, and S—setting (Nyaloko et al., 2023). The following research question was then formulated: “What is the best available evidence on strategies to enhance the cultural competence of undergraduate nursing students at nursing education institutions?”

## Sampling Process

A systematic sampling process based on the two steps described below was followed.

### Step 1: Scoping Search and Search Strategy

**Figure 1.**
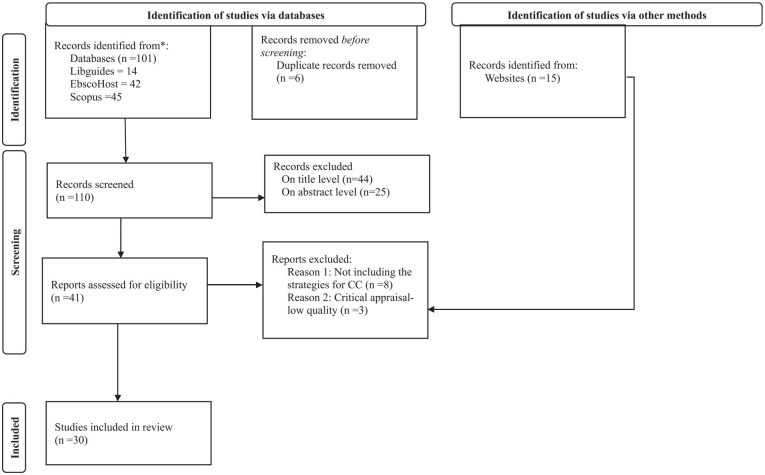
PRISMA 2020 Flow Diagram (Page et al., 2020).

The inclusion criteria were developed systematically to ensure that relevant and recent literature was included. The inclusion criteria were literature that was relevant to the cultural competence of undergraduate nursing students, written in English, available in full text, and published between 2014 and 2023. The exclusion criteria were literature that was older than 10 years (i.e., published before 2014), gray literature (e.g., research reports), and that which did not address cultural competence strategies for undergraduate nursing students’ education. Databases for the search process included EBSCOhost, Scopus, Google Scholar, and University X Libguides which included CINAHL and PubMed databases. A librarian at the university was consulted to assist in the initial search process and enhance the credibility of the process. The search string included a combination of keywords: (cultural competence AND nursing students or student nurses AND strategies or measures AND enhance or improve AND nursing education institutions). The use of Boolean operators and a librarian ensured that the most appropriate literature was included (Lubbe et al., 2020). Initial searches yielded a vast number of results (e.g., 22,000 at one stage) and limiters in the form of inclusion criteria were then applied. The final comprehensive search yielded (*N* = 122) results which were then considered in the subsequent steps of the ILR process. The Preferred Reporting Items for Systematic reviews and Meta-Analyses (PRISMA) framework (Page et al., 2020) was used to report the outcome of the literature search and selection processes. The PRISMA flow diagram depicted in Figure 1 ensures adequate reporting and transparency and further ensures credibility of the study.

### Step 2: Screening

A manual process to screen and record the literature was conducted. Porritt et al. (2014) support two-reviewer or group review to ensure transparency and reproducibility of the literature review. The PRISMA flow diagram depicted above (Figure 1) assisted the authors to systematically *identify* literature, *screen* and determine a final number of papers to be *included*. Initial screening was completed by two authors (V.N. and L.C.), while K.D.S. reviewed papers that the first two authors had not reached consensus on for inclusion. A consensus meeting with all authors was held, in which the papers to be included and excluded, based on the inclusion and exclusion criteria, were agreed upon. During the initial screening step, duplicates were excluded (*n* = 6) and a total of (*n* = 44) papers were excluded during title screening, with (*n* = 25) being excluded during abstract screening. A further (*n* = 8) papers were excluded as they were not relevant to strategies to enhance cultural competence, and (*n* = 3) were excluded due to low quality during critical appraisal. No paper was excluded as being written in a language other than English. Following this strategy, a total of 30 papers (*N* = 30) were included for data extraction and analysis.

## Data Collection

Appraising the studies to be included in a review is important to evaluate the quality and credibility of the included literature (Porritt et al., 2014). The methodological quality of the studies was reviewed by all authors, with each paper being discussed for consensus on whether to be included or not, while being guided by the set criteria. The John Hopkins Evidence-based Appraisal Tool (Dang et al., 2022) was chosen by the authors due to its rigorous quality criteria and process, thereby reducing the likelihood of bias on the part of the authors. A total number of (*n* = 41) papers were initially included for the critical appraisal process. From that sample, seven (*n* = 7) were of high quality, 31 (*n* = 31) were of good quality, and three (*n* = 3) were of low quality. Low-quality studies were excluded, and eight (*n* = 8) were excluded due to not focusing on cultural competence strategies. The authors reached a consensus to include 30 (*n* = 30) papers. Table 1 outlines the studies reviewed with their respective quality ratings.

**Table 1.** Critical Appraisal of Articles Included.

Included studies	Critical appraisal tool	Quality rating	Evidence level
1. Antón-Solanas et al. (2020). The teaching and learning Cultural Competence in a Multicultural Environment (CCMEn) model. *Nursing reports, 10*, 154–163	John Hopkins CA Tool	Good	V
2. Antón-Solanas, Tambo-Lizalde, et al. (2021). Nursing students’ experience of learning cultural competence. *PLOS ONE*, *16*(12), Article e0259802	John Hopkins CA Tool	Good	III
3. Antón-Solanas, Huércanos-Esparza, et al. (2021). Nursing lecturers’ perception and experience of teaching cultural competence: A European qualitative study. *International Journal of Environmental Research and Public Health*, *18*, 1357	John Hopkins CA Tool	Good	III
4. Antwi et al. (2019). The integration of intercultural competence in innovative pedagogical methodology in nursing education. *Abstract Proceedings International Scholars Conference, 7*(1), 193–206	John Hopkins CA Tool	Good	III
5. Byrne (2020). Evaluating cultural competence in undergraduate nursing students using standardized patients. *Teaching and Learning in Nursing, 15*, 57–60	John Hopkins CA Tool	Good	I
6. Chen et al. (2018). Evaluating student cultural competence in an associate in science in nursing program. *Teaching and Learning in Nursing, 13*, 161–167	John Hopkins CA Tool	Good	I
7. Choi & Han (2020). Effects of the global multicultural nursing competency enhancement program on the cultural competence of nursing college students. *International Journal of Advanced Nursing Education and Research, 5*(1), 43–54	John Hopkins CA Tool	Good	I
8. Choi & Kim (2018). Effects of cultural education and cultural experiences on the cultural competence among undergraduate nursing students. *Nurse Education in Practice*, 159–162	John Hopkins CA Tool	Good	III
9. Chung & Jarvill (2019). Improving nursing student cultural competence: Comparing simulation to case-based learning. *Journal of Nursing Education and Practice, 9*(7), 128–132	John Hopkins CA Tool	Good	I
10. Curtis et al. (2016). Enhancing cultural competency. *Online Journal of Cultural Competence in Nursing and Healthcare, 6*(1), 1–13	John Hopkins CA Tool	Good	I
11. Cruz et al. (2016). Predictors of cultural competence among nursing students in the Philippines: A cross-sectional study. *Nurse Education Today, 46*, 121–126	John Hopkins CA Tool	Good	III
12. Day & Beard (2019). Meaningful inclusion of diverse voices: The case for culturally responsive teaching in nursing education. *Journal of Professional Nursing, 35*(2019), 277–28	John Hopkins CA Tool	High	V
13. Debrew & Lewallen (2014). Outsiders in nursing education: Cultural sensitivity in clinical education. *Journal of Professional Nursing, 30*(2), 149–154	John Hopkins CA Tool	Good	V
14. Farokhzadian et al. (2022). Using a model to design, implement, and evaluate a training program for improving cultural competence among undergraduate nursing students: A mixed methods study. *BMC Nursing, 21*(85), 1–17	John Hopkins CA Tool	Good	III
15. Fung et al. (2023). Virtual simulation and problem-based learning enhance perceived clinical and cultural competence of nursing students in Asia: A randomized controlled cross-over study. *Nurse Education Today*	John Hopkins CA Tool	B—Good	I
16. Garneau & Pepin (2015). A constructivist theoretical proposition of cultural competence development in nursing. *Nurse Education Today, 35*, 1062–1068	John Hopkins CA Tool	A/B High/Good	III
17. Ho & Oh (2022). Development and evaluation of cultural competence course on undergraduate nursing students in Vietnam. *International Journal of Environmental Research and Public Health, 19*, 1–13	John Hopkins CA Tool	B Good	I
18. Hovland & Johannessen (2020). Nursing students develop cultural competence during student exchanges in Tanzania. *Sykepleien Forskning*	John Hopkins CA Tool	A/B High/Good	III
19. Hultsjö et al. (2019). “Cultural awareness requires more than theoretical education”—Nursing students’ experiences. *Nurse Education in Practice*	John Hopkins CA Tool	A/B High/Good	III
20. Kessler & Kost (2021). An innovative approach for using cross-cultural, collaborative simulation during undergraduate nursing study abroad exchanges. *Clinical Simulation in Nursing, 61*, 14–22	John Hopkins CA Tool	B Good	II
21. Khatib & Hadid (2019). Developing cultural competence as part of nursing studies: Language, customs and health issues. *International Journal of Studies in Nursing, 4*(1), 63–72	John Hopkins CA Tool	C Low	III
22. Kohlbry (2016). The impact of international service-learning on nursing students’ cultural competency. *Journal of Nursing Scholarship, 48*(3), 303–311	John Hopkins CA Tool	A High	III
23. Kula et al. (2021). Educating nursing students for cultural competence in emergencies: A randomized controlled trial. *BMC Nursing, 20*(184), 1–12	John Hopkins CA Tool	A High	I
24. Lin et al. (2015). Cultural competence course for nursing students in Taiwan: A longitudinal study. *Nurse Education Today*, 1268–1274	John Hopkins CA Tool	A High	II
25. Lonneman (2015). Teaching strategies to increase cultural awareness in nursing students. *Nurse Educator, 40*(6), 285–288	John Hopkins CA Tool	B Good	II
26. Markey et al. (2021). Embracing classroom cultural diversity: Innovations for nurturing inclusive intercultural learning and culturally responsive teaching. *Teaching and Learning in Nursing, 16*, 258–262	John Hopkins CA Tool	B Good	V
27. McEwing (2020). Delivering culturally competent care to the lesbian, gay, bisexual, and transgender (LGBT) population: Education for nursing students. *Nurse Education Today*	John Hopkins CA Tool	C Low	II
28. Mhlongo (2016). Cultural competency in South Africa: A nursing education perspective. *Research on Humanities and Social Sciences, 6*(16), 135–145	John Hopkins CA Tool	B Good	V
29. Mula et al. (2023). Nursing cultural competence in Israel: Does practice make it better? *Nursing Science Quarterly, 36*(1), 76–84	John Hopkins CA Tool	B Good	II
30. Ndiwane et al. (2014). Implementing standardized patients to teach cultural competency to graduate nursing student. *Journal of Clinical Nursing, 10*(2), 87–94	John Hopkins CA Tool	B Good	II
31. Noble et al. (2014). The Effect of a cultural competence educational intervention for first-year nursing students in Israel. *Journal of Transcultural Nursing, 25*(1), 87–94	John Hopkins CA Tool	B Good	II
32. O’Brien et al. (2021). Intercultural readiness of nursing students: An integrative review of evidence examining cultural competence educational interventions. *Nurse Education in Practice, 50*	John Hopkins CA Tool	B Good	V
33. Park et al. (2019). Effects of a cultural nursing course to enhance the cultural competence of nursing students in Korea. *J Educ Eval Health Prof, 16*(39)	John Hopkins CA Tool	B Good	II
34. Qin & Chaimongkol (2021). Simulation with standardized patients designed as interventions to develop nursing students’ cultural competence: A systematic review. J*ournal of Transcultural Nursing, 32*(6), 778–789	John Hopkins CA Tool	B Good	V
35. Repo et al. (2017). The cultural competence of graduating nursing students. *Journal of Transcultural Nursing, 28*(1), 98–107	John Hopkins CA Tool	B Good	II
36. Rodriguez (2020). The impact of service learning on associate degree nursing students’ cultural competence. *Creative Nursing*, 26(3), e77–e81	John Hopkins CA Tool	B Good	II
37. San (2020). The influence of the oncology-focused transgender-simulated patient simulation on nursing students’ cultural competence development. *Nursing Forum, 55*, 621–630	John Hopkins CA Tool	B Good	II
38. San (2018). Comparative analysis of cultural competence of senior and junior nursing students. *Sri Lanka Journal of Health Research, 1*(1), 14–19	John Hopkins CA Tool	B Good	II
39. Tosun (2021). Addressing the effects of transcultural nursing education on nursing students’ cultural competence: A systematic review. *Nurse Education in Practice, 55*	John Hopkins CA Tool	B Good	II
40. Turkelson et al. (2021). Using simulation to enhance nurse practitioner students cultural sensitivity, communication, and empathy with vulnerable populations. *Clinical Simulation in Nursing, 56*, 108–116	John Hopkins CA Tool	C Low quality	II
41. Ulvund & Mordal (2017). The impact of short-term clinical placement in a developing country on nursing students: A qualitative descriptive study. *Nurse Education Today, 55*, 96–100	John Hopkins CA Tool	B Good	III

## Data Analysis

### Data Extraction

A standardized tool was used to extract data. Lubbe et al. (2020) argue that to allow for data analysis, the final list of selected documents must be read thoroughly, following which the purpose, design, population, and findings must be summarized with a data extraction tool. This process was undertaken by all authors with a focus on answering the research question (see Table 2). The authors decided to use the tested data extraction forms to ensure that relevant data is extracted; this process assisted the authors to minimize bias and other research errors (Munn et al., 2014).

**Table 2:** Sample of Data Extraction.

Author(s)	Aim/purpose	Research design	Population (sample, sample size, and setting)	Findings
Antón-Solanas, Tambo-Lizalde, et al. (2021)	This paper aims to analyze European student nurses’ experience of learning cultural competence and of working with patients from diverse cultural backgrounds	Qualitative	7 semi-structured focus groups with 5–7 students took place at the participants’ respective universities in Spain, Belgium, Turkey, and Portugal	Learning cultural competence as part of curriculum; student exchange program with cultural immersion experience
Antón-Solanas, Huércanos-Esparza, et al. (2021)	The aim was to investigate nursing lecturers’ perception and experience of teaching cultural competence in four undergraduate nursing programs	Qualitative	Semi-structured personal interviews were held with a sample of 24 lecturers from four European universities	Learning in a multicultural environment; Integrating cultural competence in the curriculum
Antwi et al. (2019)	To determine the relationship between intercultural competence and the use of pedagogical methodologies	Quantitative	30 nurse educators in three universities in Trinidad and Tobago	This implies that in order to enhance the quality of nursing education, nurse educators should possess intercultural competence in order to provide innovative pedagogical methods that would enhance teaching quality and facilitate cultural diversity in the classrooms
Byrne (2020)	The purposes of this study were to describe the baseline level of self-reported cultural competence of undergraduate nursing students and compare between groups learning this material with lecture only and those learning with lecture and simulation with culturally diverse standardized patients	Mixed-method design	Convenience sample of 38 undergraduate sophomore-level nursing students in a medium-sized, religious-based university on the East Coast	Suggests that the use of standardized patients is an effective teaching strategy in nursing education particularly as a supplement to traditional lecture
Chen et al. (2018)	To evaluate student development of cultural competence over time and to identify factors that influence the development of cultural competence in an associate in science in nursing (ASN) program	Quantitative	161 ASN students included 40 students in the first semester, 40 students in the second semester, 40 students in the third semester, and 41 students in the fourth semester. Random selection of participants	Identifying the level of student cultural competence is a starting point to help faculty design teaching strategies and incorporate topics relevant to cultural diversity into learning. Inviting diverse lecturers presenting in the class to share their cultures may also facilitate students’ cultural knowledge and promote their willingness and desire to approach diversity
Choi & Kim (2018)	This study evaluated the effects of students’ personal experiences in other cultures on cultural competence and identified the respective effects of nursing students’ cultural education and personal experiences in other cultures on their cultural competence	Quantitative	236 Korean nursing students attending four universities in the Seoul, Incheon, and Gyeonggi provinces were selected through convenience sampling	Therefore, encouraging extracurricular activities in which nursing students experience and have constant contact to other cultures may be a feasible alternative to overseas travel
Chung & Jarvill (2019)	The purpose of this study was to compare the effect of simulation to case-based learning on nursing students’ perceived cultural competence, awareness, and sensitivity	Quantitative	80 baccalaureate nursing students were randomly assigned to a simulation experience or case-based learning exercise	Integration of cultural learning opportunities into nursing education is essential to provide a foundation for continued development of cultural competence. Both simulation and case-based learning improved nursing student perceived cultural awareness and sensitivity and case-based learning improved nursing student perceived cultural competence
Cruz et al. (2016)	This study investigated the predictors of cultural competence among nursing students in the Philippines	Quantitative	Convenience sample of 332 Bachelor of Science in Nursing (BSN) students registered in three Colleges of Nursing in the northern Philippines participated in this study	Cultural diversity and cultural competence should be incorporated in both classroom and clinical courses of the students throughout the nursing program to ensure a continuous development of their cultural competence. Assessment of cultural competence development among students should also be done regularly. Cultural emersion and other strategies that can provide experience for cross-cultural encounters among the students should also be adapted in the curriculum
Curtis et al. (2016)	The purpose of this study was to evaluate the use of contemporary literature and cultural learning activities in an undergraduate nursing curriculum	Quantitative	A convenience sample of 56 accelerated undergraduate nursing students	The integration of contemporary literature and Cultural learning activities across several courses provides an accessible and effective method of integrating and teaching cultural competency in a nursing curriculum
Day & Beard (2019)	This paper presents an argument for the importance of replacing the single, dominant voice in nursing education with culturally responsive teaching and offers strategies nurse educators can use to encourage students to share alternative perspectives and engage in alternative methods of discourse and communication	Expert opinion	Not applicable	The authors assert that the principles of CRT (culturally responsive teaching; Culturally mediated instruction; Teacher as facilitator; Empowering environment), adapted from teacher-education, are intuitive and can be used to empower students to voice different perspectives
Farokhzadian et al. (2022)	This study aimed to design, implement, and evaluate a cultural care-training program to improve cultural competence of undergraduate nursing students	Mixed-method study	Study setting was Razi School of Nursing and Midwifery; A conventional qualitative study was conducted—18 participants were interviewed using purposive sampling. In the second and third steps, literature review and the classic Delphi technique were used for initiation and finalization of the program. The fourth, fifth, and sixth steps were completed by implementing, monitoring, and evaluating the cultural care program (five 2-hr sessions) among 73 nursing students using a quasi-experimental design	This training program will be effective if students’ learning needs, appropriate assignments, and acceptable teaching methods are addressed. Therefore, nurse educators can design comprehensive training programs to improve nursing students’ cultural competence in different cultures and contexts. This training program is highly efficient because it is applicable in many disciplines of nursing education
Fung et al. (2023)	To compare the effectiveness of virtual simulation and PBL on the perceived clinical and cultural competence for nursing students	Quantitative	61 undergraduate and postgraduate nursing students from five Asian regions, random selection	The results revealed that students in both virtual simulation and problem-based learning had significant improvements in clinical competence questionnaire total score, nursing professional behavior, advance nursing skills after the two interventions
Garneau & Pepin (2015)	The purpose of this study was to develop a theoretical proposition of cultural competence development in nursing from a constructivist perspective	Grounded theory design	24 participants (13 nurses and 11 students) working in three community health settings in Canada	The core category, “learning to bring the different realities together to provide effective care in a culturally diverse context,” was constructed using inductive qualitative data analysis. This core category encompasses three dimensions of cultural competence: “building a relationship with the other,” “working outside the usual practice framework,” and “reinventing practice in action.”
Ho & Oh (2022)	The purpose of this study was to develop a cultural competence course and to evaluate the effects of the course on undergraduate nursing students in Vietnam	A concurrent triangulation mixed-methods study	Sixty-six nursing students, Vietnam were randomly assigned to control and experimental groups	Study support evidence that the incorporation of cultural competency into nursing education curricula enhances the level of cultural competence in undergraduate nursing students
Hovland & Johannessen (2020)	The objective of the study was to gain an insight into nursing students’ personal experiences with developing cultural competence during a 3-month exchange in Tanzania	Qualitative	25 Norwegian nursing students who were on a 3-month exchange in Tanzania, focus groups conducted	Three main themes central to development of cultural competence are (1) Receiving an explanation, (2) Maintaining an open attitude, and (3) Going outside your comfort zone Communication and interaction with people who had a different cultural background to their own supported this process. Receiving explanations of aspects they found difficult to understand contributed to the development of their cultural competence
Kessler & Kost (2019)	The aims of this paper are to describe the innovative process that occurred between students from two universities in the US and Thailand, lessons learned, and research outcomes from a pilot study for improving confidence and cultural competence to provide care and work with others from another culture.	Quantitative/qualitative design with pre-test/post-test assessment was used	35 undergraduate students (nursing, public health and health science) from US and Thailand but were on exchange in South Africa	Students’ confidence increased significantly following the cross-cultural simulation. Qualitative analysis revealed successful opportunity to collaborate cross-culturally. Study abroad simulation experiences are an effective pedagogy for bringing culturally different students together to increase knowledge, support confidence, increase cultural competence, and learn to work with others from a different culture.
Kohlbry (2016)	This article reports research findings on the effect of an international immersion service-learning project on the level and components of cultural competence of baccalaureate (BSN) nursing students	A triangulated method using quantitative pre- and post-trip surveys and a qualitative questionnaire post-immersion experience were utilized to measure cultural competency and cultural self-efficacy	The sample of 121 BSN nursing students was gathered from three southern California universities	Cultural competency is a process that nursing education must initiate with effective teaching strategies such as international service-learning immersion experiences
Lin et al. (2015)	To evaluate the effects of a cultural competence course for nursing students	Quasi-experimental longitudinal study design	105 BSN students from two universities in Taiwan, assigned to control and experiment groups	Cultural competence of all participants had improved at the posttest assessment after a two-credit course on cultural competence care. However, the overall effectiveness of the training diminished with time. The study supports that taking a cultural competence course effectively enhances the cultural competence of nursing students for a limited period of time immediately following the course
Mhlongo (2016)	The overall aim of this paper is to present an argument in support of the development of nursing education that is culturally appropriate to the South African context	Expert opinion paper	Not applicable	Nursing education curricula need to focus on providing students with the opportunity (theoretical and practical) to work in a multidisciplinary health environment. Nursing educators should ensure that they receive ongoing education and training in culturally and linguistically appropriate service delivery
Mula et al. (2023)	This study sought to measure the effect of clinical experiences with patients from diverse backgrounds on students’ cultural competence	Quantitative	300 undergraduate nursing students at the Tel Aviv–Yafo Academic College	The findings showed that nursing students’ cultural competencies were highest immediately following the academic courses on cultural competency
Ndiwane et al. (2014)	To evaluate student perceptions of their cultural competence	Quantitative	29 first-year students enrolled in an accelerated graduate nursing program located in the Northeast United States	The use of an OSCE provides a positive learning experience that adds to student comfort in working with patients from a variety of cultural backgrounds. This experience allows students to develop cross-cultural skills and increased confidence before encounters in clinical settings
Noble et al. (2014)	The purpose of this study was to evaluate the effectiveness of an educational intervention to improve the cultural competence of first-year nursing students	Quasi-experimental study	146 first-year nursing students enrolled in the Introduction to Nursing course at three different nursing schools in Israel	Our cultural competence educational intervention consisted of a faculty lecture and student group presentations. The lecture and presentations addressed cultural disparities between the patients, families, the cultures of the health care system, and the individual health care professional
O’Brien et al. (2021)	This integrative review synthesized international research that focused on educational interventions used to prepare student nurses to care for culturally diverse patients	Integrative review	14 studies were reviewed	Engaging student nurses in learning activities that augment their understanding of, and commitment to, providing culturally competent care must include a variety of integrated culturally responsive pedagogical approaches made explicit and continuously developed across all learning opportunities
Park et al. (2019)	This study aimed to verify the effect of a cultural nursing course on nursing students’ cultural competence	Interventional study with a single-group pre- and post-comparison study	62 nursing students at Dongyang University, Yeongju, Korea who attended and completed the “Multicultural Society and Nursing” course as an elective course in their major in 2015	After completing the cultural nursing course, students’ total cultural competence scores increased
Qin & Chaimongkol (2021)	This systematic review was to identify current evidence on the use of simulations with standardized patients as learning interventions that have been developed to improve nursing students’ cultural competence and on the effectiveness of those interventions	Systematic review	10 studies	Simulation with SPs increased levels of nursing students’ cultural competence. Simulations with SPs were used in theoretical and practicum courses of nursing students’ cultural competence, often combined with case-study and video presentations. Other forms of cultural education also improved cultural competence. Combination of lecture, case-based learning, and simulation with SPs can increase nursing students’ cultural competence
Repo et al. (2017)	The purpose of this study was to evaluate the level of cultural competence of graduating nursing students, to identify associated background factors to cultural competence Furthermore, to establish whether teaching multicultural nursing was implemented in nursing education	A structured Cultural Competence Assessment Tool was used in a correlational design	A sample of 295 nursing students in four polytechnics located in southern Finland	To improve cultural competence in students, nursing education should provide continuous opportunities for students to interact with different cultures, develop linguistic skills, and provide possibilities for internationalization both at home and abroad
Rodriquez (2020)	The purpose of the study was to assess the impact of an international service-learning experience on ADN students’ cultural competence	Quantitative	A convenience sample of 20 third-semester ADN students at the Point Loma Nazarene University School of Nursing signed up	Results indicated a positive effect between participating in an international service-learning experience and increased levels of cultural competence. International service learning provides ADN students the opportunity to incorporate classroom learning into their care of populations living within diverse communities
San (2019)	It aimed to improve students’ knowledge, skills, and attitudes with regard to providing culturally competent nursing care	Quantitative	53 nursing students, United states Convenience sampling	The Diverse Standardized Patient simulation (DSPS) influenced statistically significant changes (increase) in students’ trans-cultural self-efficacy perceptions (*p* < .05). All students regardless of background benefited from formalized cultural competence education
Tosun et al. (2021)	This study aimed to synthesize the findings of studies evaluating educational programs providing curricular transcultural nursing education	Systematic review	11 research papers	Different durations and types of teaching methods included debates, discussions, case scenarios, practicums, simulation, international learning projects, experiential learning, storytelling, and traditional teaching lectures. In 10 studies, an increase in the level of culture-related competences was reported as statistically significant
Ulvund & Mordal (2017)	To investigated how short-term international clinical placement impacted Norwegian nursing students’ development of cultural competency	Qualitative	18 undergraduate nursing, Ethiopia students who experienced 4 weeks of clinical placement in Ethiopia	Findings suggested that real-life experience culturally awakened the students and forced an ongoing process developing cultural competence. However, it is important to give students time to reflection

### Data Synthesis and Thematic Analysis

Data synthesis and thematic analysis followed the extraction phase. Data synthesis was conducted concurrently with thematic analysis whereby patterns within the data were analyzed and reported (Braun & Clarke, 2006). The authors applied the six steps of thematic analysis by Braun and Clarke (2006) which included familiarizing themselves with the extracted data; generating initial codes; searching for themes; reviewing themes; defining and naming themes; and finally, writing up the report. This process was followed until the data in Table 3 were formulated. The systematic process followed allowed the authors to reach consensus on the themes reported.

**Table 3.** Thematic Synthesis Table to Summarize New Constructs Derived From Good Quality Documents.

Themes	Subthemes	No.	Studies that informed themes
1. Integrating cultural competence into the undergraduate nursing curriculum	• Integrating cultural competence in the curriculum • Design teaching strategies and incorporate topics relevant to cultural diversity into learning • Integration of cultural learning opportunities into nursing education • Cultural diversity and cultural competence should be incorporated in both classroom and clinical courses • Integration of contemporary literature and cultural learning activities • Incorporation of cultural competency into nursing education curricula • Course on cultural competence care • Academic courses on cultural competency • Cultural competence educational intervention consisted of a faculty lecture and student group presentations • Cultural nursing course	12	Antón-Solanas, Tambo-Lizalde, et al. (2021), Antón-Solanas, Huércanos-Esparza, et al. (2021), Chen et al. (2018), Chung & Jarvill (2019), Cruz et al. (2016), Curtis et al. (2016), Ho & Oh (2022), San (2019), Lin et al. (2015), Mula et al. (2023), Noble et al. (2014), Park et al. (2019)
2. Cultural immersion	• Student exchange program with cultural immersion experience • Learning in a multicultural environment • Extracurricular activities in which nursing students experience and have constant contact to other cultures • Cultural emersion • Communication and interaction with people who had a different cultural background • Effective teaching strategies such as international service-learning immersion experiences • Provide continuous opportunities for students to interact with different cultures, develop linguistic skills, and provide possibilities for internationalization both at home and abroad • International service-learning experience	12	Antón-Solanas, Tambo-Lizalde, et al. (2021), Antón-Solanas, Huércanos-Esparza, et al. (2021), Antwi et al. (2019), Choi & Kim (2018), Cruz et al. (2016), Kessler & Kost (2019), Mhlongo (2016), Hovland & Johannessen (2020), Ulvund & Mordal (2017), Rodriquez (2020), Repo et al. (2017), Kohlbry (2016)
3. Innovative pedagogical approaches	• Provide innovative pedagogical methods • Simulation and case-based learning • Standardized patients is an effective teaching strategy • Use of principles of CRT (culturally responsive teaching) • Design comprehensive training programs • Virtual simulation and problem based learning • “Building a relationship with the other,” “working outside the usual practice framework,” and “reinventing practice in action.” • Cross-cultural simulation • The use of an OSCE provides a positive learning experience • A variety of integrated culturally responsive pedagogical approaches made explicit and continuously developed across all learning opportunities • Simulation with SPs increased levels of nursing students’ cultural competence • Diverse Standardized Patient simulation (DSPS) • Real-life experience culturally awakened the students • Reflection is important	14	Antwi et al. (2019), Byrne (2020), Chung & Jarvill (2019), Day & Beard (2019), Fung et al. (2023), Kessler & Kost (2019), San (2019), Qin & Chaimongkol (2021), O’Brien et al. (2021), Noble et al. (2014), Farokhzadian et al. (2022), Garneau & Pepin (2015), Ndiwane et al. (2014), Ulvund & Mordal (2017)
4. Role of nurse educators	• Nurse educators should possess intercultural competence • Inviting diverse lecturers • Teacher as facilitator • Nursing educators should ensure that they receive ongoing education and training in culturally and linguistically appropriate service delivery • Different durations and types of teaching methods	6	Antwi et al. (2019), Chen et al. (2018), Day & Beard (2019), Farokhzadian et al. (2022), Mhlongo (2016), Tosun et al. (2021)
5. Students’ assessments	• Identifying the level of student cultural competence • Assessment of cultural competence development	2	Chen et al. (2018), Cruz et al. (2016)

## Findings

Thirty (*n* = 30) papers were included in the review process. Of these, nine (*n* = 9) were non-experimental quantitative studies, six (*n* = 6) quantitative experimental and intervention studies, five (*n* = 5) qualitative studies, five (*n* = 5) mixed-method studies, three (*n* = 3) review studies, and two (*n* = 2) expert opinion papers. The empirical research papers included reported on the findings of studies conducted in the United States of America (*n* = 9), Korea (*n* = 3), and Israel (*n* = 2). The following countries had one paper each: Ethiopia, Taiwan, Finland, Norway, Vietnam, Canada, Iran, Philippines, and Trinidad and Tobago. There were four multi-country studies with participants from Spain, Belgium, Turkey, and Portugal (*n* = 2), Hong Kong, Mainland China, Thailand, Korea, and Taiwan (*n* = 1), and the United States of America and Thailand (*n* = 1).

Thematic analysis of the extracted data revealed five strategies to enhance the cultural competence of undergraduate nursing students at NEIs, which are reported in the following sections.

## Strategy 1—Integrating Cultural Competence Into the Undergraduate Nursing Curriculum

Integrating cultural competency into the undergraduate nursing curricula, as supported in 12 papers (Antón-Solanas, Huércanos-Esparza, et al., 2021; Antón-Solanas, Tambo-Lizalde, et al., 2021; Chen et al., 2018; Chung & Jarvill, 2019; Cruz et al., 2016; Curtis et al., 2016; Ho & Oh, 2022; Lin et al., 2015; Mula et al., 2023; Noble et al., 2014; Park et al., 2019; San, 2019), covers a variety of curricula-related interventions to enhance the cultural competence of students. This includes designing teaching strategies that enhance cultural competencies, and incorporating topics related to cultural diversity into subject content and their activities (Chen et al., 2018). Educational interventions to enhance cultural competency were reported to include faculty and student group presentations (Noble et al., 2014), and the use of contemporary literature on cultural competency (Curtis et al., 2016). However, Cruz et al. (2016) indicated that for this to be effective, cultural competence should be incorporated in both clinical training and theory courses. San (2019) highlighted that nursing students benefit from formalized cultural competence education. Formalization can be achieved by the design and implementation of culturally competent care courses for nursing students (Lin et al., 2015; Mula et al., 2023; Park et al., 2019).

## Strategy 2—Cultural Immersion

To enhance cultural competence in undergraduate nursing students, as identified in 12 papers (Antón-Solanas, Huércanos-Esparza, et al., 2021; Antón-Solanas, Tambo-Lizalde, et al., 2021; Antwi et al., 2019; Choi & Kim, 2018; Cruz et al., 2016; Hovland & Johannessen, 2020; Kessler & Kost, 2021; Kohlbry, 2016; Mhlongo, 2016; Repo et al., 2017; Rodriguez, 2020; Ulvund & Mordal, 2017), culturally immersive experiences should be provided. Cultural immersion is important as it provides students with exposure to different patient backgrounds and encourages learning to take place in consideration of cultural diversity. Cultural immersion reported in the included papers provided learning in multicultural environments (Antón-Solanas, Tambo-Lizalde, et al., 2021; Mhlongo, 2016), allowing for cross-cultural encounters for both theoretical and practical learning (Cruz et al., 2016), and communication and interaction between students and culturally diverse individuals (Hovland & Johannessen, 2020). Interactions with different cultures help students to develop their basic linguistic skills (Repo et al., 2017) and provide an opportunity for ongoing training and education in culturally appropriate health care delivery (Mhlongo, 2016).

Furthermore, the reviewed studies reported cultural immersion through student exchange programs which included studying abroad or international placements for a specified period of time (Antón-Solanas, Tambo-Lizalde, et al., 2021; Kessler & Kost, 2021; Kohlbry, 2016; Repo et al., 2017; Rodriguez, 2020). Some student exchange programs incorporated simulation experiences with culturally diverse students to promote collaborative learning (Kessler & Kost, 2021) and international service learning (Kohlbry, 2016; Rodriguez, 2020). Other culturally immersive experiences reported were the facilitation of cultural diversity in the classroom (Antwi et al., 2019) and extracurricular activities with an option to have constant contact with other cultures (Choi & Kim, 2018).

## Strategy 3—Innovative Pedagogical Approaches

The reviewed studies reported innovative pedagogical approaches used to enhance cultural competency in undergraduate nursing students (Antwi et al., 2019; Byrne, 2020; Chung & Jarvill, 2019; Day & Beard, 2019; Farokhzadian et al., 2022; Fung et al., 2023; Garneau & Pepin, 2015; Kessler & Kost, 2021; Ndiwane et al., 2014; Noble et al., 2014; O’Brien et al., 2021; Qin & Chaimongkol, 2021; San, 2019; Ulvund & Mordal, 2017). Simulation as a pedagogical approach was reported by Chung and Jarvill (2019), Fung et al. (2023), Kessler and Kost (2021), Qin and Chaimongkol (2021), and San (2019). Simulation was conducted in face-to-face encounters and virtually. In some cases, simulation was conducted together with other approaches such as case-based study and problem-based solving to enhance its effectiveness and impact on student learning. Qin and Chaimongkol (2021) reported that simulation with standardized patients in theoretical and practical courses increased student cultural competence. While standardized patients may be from similar backgrounds to students, diverse standardized patients provide positive outcomes when embedded in formalized cultural competence education (San, 2019). Standardized patients were used in some educational contexts as a supplement to traditional teaching (Byrne, 2020). For an improved cultural competency learning experience, detailed preparation and immediate feedback after simulation encounters should be provided (Ndiwane et al., 2014).

In addition, comprehensive training programs were followed to improve nursing students’ cultural competency (Farokhzadian et al., 2022), with opportunities to engage students in learning activities that promote critical thinking and opportunities to reflect. Some included objective structured clinical examinations (OSCE) in students’ cultural learning experiences to provide opportunity to practice asking culturally appropriate questions (Ndiwane et al., 2014). This increased student confidence and facilitated the development of cross-cultural skills in preparation for encounters with real patients. In some contexts, examples and case studies were used to illustrate the theory of cultural competence (Antón-Solanas, Huércanos-Esparza, et al., 2021). A grounded theory study reported that students became culturally competent through building relationships with others, working outside the usual practice context, and reinventing practice in action (Garneau & Pepin, 2015). In some contexts, educators considered culturally responsive teaching to consist of elements such as culturally mediated instructions and an empowering environment (Day & Beard, 2019).

## Strategy 4—Role of Nurse Educators

Six studies provided evidence that nurse educators (NEs) play a role in enhancing the cultural competency of nursing students (Antwi et al., 2019; Chen et al., 2018; Day & Beard, 2019; Farokhzadian et al., 2022; Mhlongo, 2016; Tosun et al., 2021). The NE is a facilitator of cultural competence education and has a mandate to design comprehensive training programs that are responsive to student learning needs, with appropriate learning activities and acceptable teaching techniques (Farokhzadian et al., 2022). Training programs can be of different duration and make use of different types of teaching techniques to increase cultural competence (Tosun et al., 2021). The variety of teaching techniques reported included storytelling, case discussions, simulations, practicum, traditional lecture method, case scenarios, discussions, debate, international learning projects, and experiential learning experiences. To facilitate innovative pedagogical approaches, NEs should possess intercultural competence (Antwi et al., 2019). Thus, they should be able to facilitate cultural diversity through quality classroom teachings. NEs have a role to invite diverse lecturers to provide classroom presentations on their own culture; this practice increases students’ cultural knowledge and, in a way, facilitates their desire and willingness to work with diverse individuals (Chen et al., 2018).

Moreover, to fulfill their roles in facilitating cultural competence, the review indicated that NEs need to practice cultural awareness. This can be done by appreciating that they are cultural beings themselves (Mhlongo, 2016). Mhlongo (2016) further reported that NEs should apply the theory and principles of cultural competency during their daily practice, and not only in the process of teaching. Consequently, NEs have a responsibility to make sure they obtain necessary continuous training and education for culturally and linguistically appropriate service delivery.

## Strategy 5—Students’ Assessment

The findings of the reviewed studies demonstrate that the assessment of students has a role in enhancing cultural competence (Chen et al., 2018; Cruz et al., 2016; Ndiwane et al., 2014). Findings from Chen et al. (2018) indicated that for NEs to design relevant topics and teaching pedagogies relevant for cultural diversity in learning, identifying the levels of cultural competence in students should be the starting point. In addition, assessing students via OSCE with standardized patients provides feedback on professionalism, communication skills, history taking, and closure of interviews (Ndiwane et al., 2014). Cruz et al. (2016) recommended regular assessment of students for the monitoring and evaluation of their cultural competence development.

## Discussion

The objective of this ILR was achieved as the strategies that can be used to enhance the cultural competence of undergraduate nursing students at NEIs were outlined. Five themes emerged as strategies to enhance cultural competence: integrating cultural competence into the undergraduate nursing curriculum, cultural immersion, innovative pedagogical approaches, the role of nurse educators, and students’ assessment.

The first strategy, *integrating cultural competence into the undergraduate nursing curriculum*, included integration at the curriculum design level by designing stand-alone cultural competence courses, or by incorporating different cultural competence learning activities into existing classroom and clinical teaching programs. To prepare nursing students to be culturally competent practitioners, the nursing curriculum must be inclusive of activities that enhance culturally competent care of diverse populations (James & Al-Kofahy, 2021).

The second strategy, *cultural immersion*, focused on exposing students to or integrating their learning activities with diverse or multicultural contexts. According to Granel et al. (2021), cultural competence is developed as an outcome of both national/local and international experiences. International student exchange programs allow students to experience a new culture and develop competencies in terms of interaction skills, confidence, discovering new potentials, and increasing their understanding of global perspectives of nursing (Aldén-Joyce et al., 2023).

Some international and local culturally immersive experiences for nursing students incorporated service-learning. Due to students’ immersion in the community context through the provision of services, this approach enhances the students’ cultural competence (De Leon, 2014; Emrani et al., 2024). Wall-Basset et al. (2018) using Campinha-Bacote’s model confirmed that the use of reflective journals, service-learning projects, site exposures, and participating together during an international service learning helped deepen and progress the cultural competence process for students. Moreover, students develop cultural competence by gaining knowledge and skills to work with diverse patients. This sets the stage for developing an understanding of cultural diversities, practicing ethical decision-making, developing problem-solving and critical thinking skills, and acquiring self-efficacy in new environments (Rodriguez, 2020).

Placement in multicultural contexts is, however, associated with challenges related to language barriers, a lack of awareness of cultural practices, differing values, norms, and cultural expectations of patients, and resistance to health care interventions (Nuuyoma et al., 2024). This review demonstrates that these placements enhance cultural competence due to communication and interaction with different cultural backgrounds, thereby facilitating the development of linguistic skills and understanding of diverse cultural practices.

The third strategy, *innovative pedagogical approaches*, was supported by the majority (*n* = 14) of the papers reviewed. This strategy focuses on using teaching methods or techniques that accommodate students with a variety of learning styles, and the active involvement of students, to enhance cultural competence. Simulation as an innovative pedagogical approach in reviewed studies was conducted either in face-to-face encounters or virtually. In nursing education, simulation as a pedagogic tool allows for the application of knowledge and the advancement of clinical and critical thinking skills (Qin & Chaimongkol, 2021). When conducted virtually, NEs facilitating simulation sessions should make sure activities are appropriate for learners’ levels, and the sequencing of cases should be appropriate to facilitate the building and scaffolding of knowledge (Verkuyl & Atack, 2024). The findings of our review indicate that simulation is, in some cases, conducted together with other approaches such as case-based study, use of examples, and problem-based solving to enhance its effectiveness and impact on student learning.

NEs are encouraged to use culturally responsive teaching (CRT) as part of innovative pedagogical approaches (Day & Beard, 2019; O’Brien et al., 2021). CRT is an umbrella concept for pedagogies that prepare students to support social justice in and beyond the classroom (Day & Beard, 2019). CRT may be implemented with the guidance of three principles: culturally mediated instruction, teacher as a facilitator, and creating an empowering environment. While caring for diverse patients, students learn cultural competence by bringing different realities together to provide effective care, working outside the prescribed practice framework, and building relationships with others, especially people from diverse cultures (Garneau & Pepin, 2015). Irrespective of the CRT principle and method followed, it should be explicit, regularly updated, and integrated into all available learning opportunities (O’Brien et al., 2021). In addition, students should be allowed time to reflect on learning experiences to promote developing cultural competence (Ulvund & Mordal, 2017).

The fourth strategy, *the role of nurse educators (NEs)*, focused on the actions and mandate of NEs in enhancing the cultural competence of nursing students. This review reveals that NEs should possess intercultural competence. Rahimi et al. (2023), in agreement, state that to prepare culturally competent nursing students for clinical practice, NEs themselves should be culturally competent. Therefore, NEs should receive ongoing training and education in culturally and linguistically appropriate health care service delivery (Mhlongo, 2016). This may be conducted via short courses or formal postgraduate training courses.

The current ILR further revealed that NEs should practice cultural awareness. Literature emphasized the practice of cultural awareness and cultural desire as extensively contributing to the development of cultural competence in NEs (Majnoon et al., 2023). One of the roles of NEs in enhancing the cultural competence of undergraduate nursing students is acting as a facilitator.

The fifth strategy, *students’ assessment*, focused on how assessment may be used to enhance cultural competency in undergraduate nursing students. Through assessment, NEs can make informed decisions about a student’s level of competence and help to bridge the gap between theory and practice (Donough, 2022). Assessment is not only for monitoring and evaluation of student progress but also to guide NEs in designing appropriate teaching pedagogies and topics for cultural diversity that are responsive to identified needs (Chen et al., 2018). Moreover, this review reveals that there should be continuous assessment for students to develop cultural competence. This is relevant because cultural competence is not stagnant; rather, it is a process that can be affected by cultural context, social, historical, and political aspects, and even individuals’ immediate financial situation (Sharifi et al., 2019). NEs should use a variety of assessment methods; however, an OSCE with standardized patients is preferred as it allows feedback on aspects such as professionalism, communication skills, and generally how history taking and closure of interviews are done (Ndiwane et al., 2014).

## Implications, Strengths, and Limitations

This study’s findings have implications for nursing education and practice. The strategies identified can be used by different nursing education stakeholders to promote cultural competency education in curriculum design, implementation, and evaluation phases. The stakeholders in this context refer to educators in NEIs as well as nurse practitioners in clinical settings. Moreover, these strategies may be used to inform the development of local frameworks and guidelines to enhance the cultural competency of undergraduate nursing students. Future researchers may consider assessing the cultural competency levels of undergraduate nursing students from developing or middle-to-low-income countries and interventions to promote cultural competence in these contexts.

A strength of this ILR is the diverse quality and evidence levels of the literature reviewed. However, empirical studies were conducted outside the African continent, except for one from Ethiopia. This is considered a limitation as cultural competence tends to be influenced by political, social, historical, cultural, and financial situations.

## Conclusion

The objective of this ILR was to understand and outline strategies that can be used to enhance the cultural competence of undergraduate nursing students at NEIs. Most of the reviewed literature supported the use of innovative pedagogical approaches, the integration of cultural competence training in the undergraduate nursing curriculum, and cultural immersion of students. These strategies are foundational to enhancing cultural competence and should be supported by NEIs to ensure that appropriate pedagogy and teaching strategies are designed. The role of nurse educators is critical in the process of enhancing nursing students’ cultural competence as they are facilitators of this process. These strategies thus provide nurse educators with evidence-based information to incorporate in nursing curricula. It is recommended that nurse educators be capacitated with cultural competence skills as only then will they be able to facilitate this process for students, which should start with the development of innovative curricula focused on cultural competence.

## References

[bibr1-10436596241301407] Aldén-Joyce T. Mattson J. Scheers-Andersson E. Kidayi P. Rogathi J. Cadstedt J. Björling G. (2023). Tanzanian nursing students’ experiences of student exchange in Sweden—A qualitative case study. SAGE Open Nursing, 9, 1–10. 10.1177/23779608231160923 PMC9989424 36895707

[bibr2-10436596241301407] Almutairi A. F. Adlan A. A. Nasim M. (2017). Perceptions of the critical cultural competence of registered nurses in Canada. BMC Nursing, 16(1), 1–9. 10.1186/s12912-017-0242-2 28824334 PMC5558749

[bibr3-10436596241301407] Almutairi A. F. McCarthy A. Gardner G. E. (2015). Understanding cultural competence in a multicultural nursing workforce: Registered nurses’ experience in Saudi Arabia. Journal of Transcultural Nursing, 26(1), 16–23. 10.1177/1043659614523992 24626280

[bibr4-10436596241301407] Antón-Solanas I. Huércanos-Esparza I. Hamam-Alcober N. Vanceulebroeck V. Dehaes S. Kalkan I. Kömürcü N. Coelho M. Coelho T. Casa-Nova A. Cordeiro R. Ramón-Arbués E. Moreno-González S. Tambo-Lizalde E. (2021). Nursing lecturers’ perception and experience of teaching cultural competence: A European qualitative study. International Journal of Environmental Research and Public Health, 18, 1–20. 10.3390/ijerph18031357 PMC7908137 33540907

[bibr5-10436596241301407] Antón-Solanas I. Tambo-Lizalde E. Hamam-Alcober N. Vanceulebroeck V. Dehaes S. Kalkan I. Kömürcü N. Coelho M. Coelho T. Nova A. C. Cordeiro R. Sagarra-Romero L. Subirón-Valera A. B. Huércanos-Esparza I. (2021). Nursing students’ experience of learning cultural competence. PLOS ONE, 16(12), 1–24. 10.1371/journal.pone.0259802 PMC8683022 34919540

[bibr6-10436596241301407] Antwi F. Osei S. Peprah W. Antwi M. Antwi E. (2019). The integration of intercultural competence in innovative pedagogical methodology in nursing education. Abstract Proceedings International Scholars Conference, 7(1), 193–206. 10.35974/isc.v7i1.944

[bibr7-10436596241301407] Braun V. Clarke V. (2006). Using thematic analysis in psychology. Qualitative Research in Psychology, 3(2), 77–101. 10.1191/1478088706qp063oa

[bibr8-10436596241301407] Byrne D. (2020). Evaluating cultural competence in undergraduate nursing students using standardized patients. Teaching and Learning in Nursing, 15(1), 57–60. 10.1016/j.teln.2019.08.010

[bibr9-10436596241301407] Campinha-Bacote J. (2002). The process of cultural competence in the delivery of healthcare services: A model of care. Journal of Transcultural Nursing, 13(3), 181–184. 10.1177/10459602013003003 12113146

[bibr10-10436596241301407] Chen H. C. Jensen F. Measom G. Nichols N. D. (2018). Evaluating student cultural competence in an associate in science in nursing program. Teaching and Learning in Nursing, 13(3), 161–167. 10.1016/j.teln.2018.03.005

[bibr11-10436596241301407] Choi J. S. Kim J. S. (2018). Effects of cultural education and cultural experiences on the cultural competence among undergraduate nursing students. Nurse Education in Practice, 29, 159–162. 10.1016/j.nepr.2018.01.007 29360621

[bibr12-10436596241301407] Chung S. Jarvill M. (2019). Improving nursing student cultural competence: Comparing simulation to case-based learning. Journal of Nursing Education and Practice, 9(7), 128–132. 10.5430/jnep.v9n7p128

[bibr13-10436596241301407] Cross R. Bone E. Ampt P. Bell T. Quinnell R. Gongora J. (2020). Cultural competence and the higher education sector. In Frawley J. Russell G. Sherwood J. (Eds.), Cultural competence and the higher education sector: Australian perspectives, policies and practices (pp. 255–275). Springer. 10.1007/978-981-15-5362-2

[bibr14-10436596241301407] Cruz J. P. Estacio J. C. Bagtang C. E. Colet P. C. (2016). Predictors of cultural competence among nursing students in the Philippines: A cross-sectional study. Nurse Education Today, 46, 121–126. 10.1016/j.nedt.2016.09.001 27636832

[bibr15-10436596241301407] Curtis M. Bultas M. Green L. (2016). Enhancing cultural competency. Online Journal of Cultural Competence in Nursing and Healthcare, 6(1), 1–13. 10.9730/ojccnh.org/v6n1a1

[bibr16-10436596241301407] Dang D. Dearholt S. Bissett K. Ascenzi J. Whalen M . (2022). Johns Hopkins evidence-based practice for nurses and healthcare professionals: Model and guidelines. (4th ed.) Sigma Theta Tau International.

[bibr17-10436596241301407] Day L. Beard K. (2019). Meaningful inclusion of diverse voices: The case for culturally responsive teaching in nursing education. Journal of Professional Nursing, 35(4), 277–281. 10.1016/j.profnurs.2019.01.002 31345507

[bibr18-10436596241301407] De Leon N . (2014). Developing intercultural competence by participating in intensive intercultural service-learning. Michigan Journal of Community Service Learning, 21, 17–30.

[bibr19-10436596241301407] Donough G. (2022). Designing theoretical assessments at nursing higher education institutions: A scoping review. South African Journal of Higher Education, 36(2), 79–98. 10.20853/36-2-4699

[bibr20-10436596241301407] Emrani M. Khoshnood Z. Farokhzadian J. Sadeghi M. (2024). The effect of service-based learning on health education competencies of students in community health nursing internships. BMC Nursing, 23(1), 1–8. 10.1186/s12912-024-01799-y 38395792 PMC10893737

[bibr21-10436596241301407] Farokhzadian J. Nematollahi M. Nayeri N. D. (2022). Using a model to design, implement, and evaluate a training program for improving cultural competence among undergraduate nursing students: A mixed methods study. BMC Nursing, 21(85), 1–18. 10.1186/s12912-022-00849-7 35410203 PMC8996203

[bibr22-10436596241301407] Fung J. Chan S. Takemura H. Chiu H. Huang H. Lee J. Preechawong S. Yuel M. Sun M. Xia W. Xiao J. Lin C. (2023). Virtual simulation and problem-based learning enhance perceived clinical and cultural competence of nursing students in Asia: A randomized controlled cross-over study. Nurse Education Today, 123, 1–7. 10.1016/j.nedt.2023.105721 36774904

[bibr23-10436596241301407] Garneau A. B. Pepin J. (2015). A constructivist theoretical proposition of cultural competence development in nursing. Nurse Education Today, 35(11), 1062–1068. 10.1016/j.nedt.2015.05.019 26077350

[bibr24-10436596241301407] Gradellini C. Gómez-Cantarino S. Dominguez-Isabel P. Molina-Gallego B. Mecugni D. Ugarte-Gurrutxaga M. I. (2021). Cultural competence and cultural sensitivity education in university nursing courses: A scoping review. Frontiers in Psychology, 12, Article 682920. 10.3389/fpsyg.2021.682920 PMC8514292 34659003

[bibr25-10436596241301407] Granel N. Leyva-Moral J. M. Morris J. Šáteková L. Grosemans J. Bernabeu-Tamayo M. D. (2021). Student’s satisfaction and intercultural competence development from a short study abroad programs: A multiple cross-sectional study. Nurse Education in Practice, 50, 1–6. 10.1016/j.nepr.2020.102926 33227616

[bibr26-10436596241301407] Ho T. T. T. Oh J. (2022). Development and evaluation of cultural competence course on undergraduate nursing students in Vietnam. International Journal of Environmental Research and Public Health, 19(2), 1–13. 10.3390/ijerph19020888 PMC8776207 35055710

[bibr27-10436596241301407] Hovland O. J. Johannessen B. (2020). Nursing students develop cultural competence during student exchanges in Tanzania. Sykepleien Forskning, 13, 1–17. 10.4220/sykepleienf.2018.73782en

[bibr28-10436596241301407] James L. Al-Kofahy L. (2021). Cultivating cultural competence through academic community engagement and clinical reflection. Journal of Transcultural Nursing, 32(5), 623–629. 10.1177/1043659620971699 33174503

[bibr29-10436596241301407] Jeffreys M.R . (2019). Evidence-based updates and universal utility of Jeffreys’ cultural competence and confidence framework for nursing education (and beyond) through TIME. Annual Review of Nursing Research, 37(1), 43–117. http://dx.doi.org/10.1891/0739-6686.37.43 10.1891/0739-6686.37.1.43 30692154

[bibr30-10436596241301407] Kessler T. A. Kost G. C. (2021). An innovative approach for using cross-cultural, collaborative simulation during undergraduate nursing study abroad exchanges. Clinical Simulation in Nursing, 61, 14–22. 10.1016/j.ecns.2021.09.004

[bibr31-10436596241301407] Kohlbry P. W. (2016). The impact of international service-learning on nursing students’ cultural competency. Journal of Nursing Scholarship, 48(3), 303–311. 10.1111/jnu.12209 27111382

[bibr32-10436596241301407] Lin C. J. Chang P. Rong Wang L. H. Huang M. C. (2015). Cultural competence course for nursing students in Taiwan: A longitudinal study. Nurse Education Today, 35(12), 1268–1274. 10.1016/j.nedt.2015.05.023 26094199

[bibr33-10436596241301407] Lubbe W. Ham-Baloyi W. t. Smit K . (2020). The integrative literature review as a research method: A demonstration review of research on neurodevelopmental supportive care in preterm infants. Journal of Neonatal Nursing, 26(6), 308–315. 10.1016/j.jnn.2020.04.006

[bibr34-10436596241301407] Majnoon S. Yates V. M. Asgarpour H. Mirza Aghazadeh Attari A. Lotfi M. (2023). Cultural competence of nursing educators at medical universities of 2nd regional planning in Iran. BMC Medical Education, 23(1), 1–12. 10.1186/s12909-023-04274-5 37170271 PMC10176968

[bibr35-10436596241301407] Mhlongo T. P. (2016). Cultural competency in South Africa: A nursing education perspective. Research on Humanities and Social Sciences, 6(16), 135–145. www.iiste.org

[bibr36-10436596241301407] Mula T. Azuri P. Baumann S. L. (2023). Nursing cultural competence in Israel: Does practice make it better? Nursing Science Quarterly, 36(1), 78–84. 10.1177/08943184221131968 36571320

[bibr37-10436596241301407] Munn Z. Tafanaru C. Aromataris E. (2014). Data extraction and synthesis: The steps following study selection in a systematic review. The Joana Briggs Institute. 10.1097/01.naj.0000451683.66447.89

[bibr38-10436596241301407] Ndiwane A. Koul O. Theroux R. (2014). Implementing standardized patients to teach cultural competency to graduate nursing students. Clinical Simulation in Nursing, 10(2), e87–e94. 10.1016/j.ecns.2013.07.002

[bibr39-10436596241301407] Noble A. Nuszen E. Rom M. Noble L. M. (2014). The effect of a cultural competence educational intervention for first-year nursing students in Israel. Journal of Transcultural Nursing, 25(1), 87–94. 10.1177/1043659613503881 24060806

[bibr40-10436596241301407] Nuuyoma V. Muvumwaeni S. Chihururu L. (2024). Transcultural nursing: A qualitative analysis of nursing students’ experiences in a multicultural context in North-Eastern Namibia. BMC Nursing, 23, 1–13. 10.1186/s12912-024-01773-8 38360601 PMC10870613

[bibr41-10436596241301407] Nyaloko M. Lubbe W. Moloko-Phiri S. S. Shopo K. D. (2023). Exploring cultural determinants to be integrated into preterm infant care in the neonatal intensive care unit: An integrative literature review. BMC Pregnancy and Childbirth, 23, Article 15. 10.1186/s12884-022-05321-7 PMC9830862 36624421

[bibr42-10436596241301407] O’Brien E. O’Donnell C. Murphy J. O’Brien B. Markey K. (2021). Intercultural readiness of nursing students: An integrative review of evidence examining cultural competence educational interventions. Nurse Education in Practice, 50, 1–16. 10.1016/j.nepr.2021.102966 33454512

[bibr43-10436596241301407] Page M. J. McKenzie J. E. Bossuyt P. M. Boutron I. Hoffmann T. C. Mulrow C. D. Shamseer L. Tetzlaff J. M. Akl E. A. Brennan S. E. Chou R. Clark J. Cumpston M. Davies P. Deeks J. J. Duffy S. Elbers R. Gtzsche P. C. Hróbjartsson A. ... Welch V . (2020). The PRISMA 2020 statement: an updated guideline for reporting systematic reviews. BMJ, 372(71). https://doi:10.1136/bmj.n71 10.1136/bmj.n71 PMC8005924 33782057

[bibr44-10436596241301407] Park H. S. Jang H. J. Jeong G. H. (2019). Effects of a cultural nursing course to enhance the cultural competence of nursing students in Korea. Journal of Educational Evaluation for Health Professions, 16(39), 1–8. 10.3352/JEEHP.2019.16.39 32299189 PMC7040427

[bibr45-10436596241301407] Porritt K. Gomersall J. Lockwood C . (2014). JBI’s Systematic Reviews: Study selection and critical appraisal. AJN, American Journal of Nursing, 114(6), p. 47–52,| DOI: 10.1097/01.NAJ.0000450430.97383.64 24869584

[bibr46-10436596241301407] Qin Y. Chaimongkol N. (2021). Simulation with standardized patients designed as interventions to develop nursing students’ cultural competence: A systematic review. Journal of Transcultural Nursing, 32(6), 778–789. 10.1177/10436596211023968 34120529

[bibr47-10436596241301407] Rahimi M. Khodabandeh Shahraki S. Fatehi F. Farokhzadian J. (2023). A virtual training program for improving cultural competence among academic nurse educators. BMC Medical Education, 23(1), Article 445. 10.1186/s12909-023-04414-x PMC10276432 37328780

[bibr48-10436596241301407] Repo H. Vahlberg T. Salminen L. Papadopoulos I. Leino-Kilpi H. (2017). The cultural competence of graduating nursing students. Journal of Transcultural Nursing, 28(1), 98–107. 10.1177/1043659616632046 26873438

[bibr49-10436596241301407] Riva J. J. Malik K. M. Burnie S. J. Endicott A. R. Busse J. W. (2012). What is your research question? An introduction to the PICOT format for clinicians. Journal of the Canadian Chiropractic Association, 56(3), 167–171. 22997465 PMC3430448

[bibr50-10436596241301407] Rodriguez E. M. (2020). The impact of service learning on associate degree nursing students’ cultural competence. Creative Nursing, 26(3), e77–e81. 10.1891/CRNR-D-20-00038 32883830

[bibr51-10436596241301407] Rukadikar C. Mali S. Bajpai R. Rukadikar A. Singh A. K. (2022). A review on cultural competency in medical education. Journal of Family Medicine and Primary Care, 11, 4319–4329. 10.4103/jfmpc.jfmpc_2503_21 PMC9638640 36352918

[bibr52-10436596241301407] San E. (2019). Effect of the diverse standardized patient simulation (DSPS) cultural competence education strategy on nursing students’ transcultural self-efficacy perceptions. Journal of Transcultural Nursing, 30(3), 291–302. 10.1177/1043659618817599 30539683

[bibr53-10436596241301407] Sharifi N. Adib-Hajbaghery M. Najafi M. (2019). Cultural competence in nursing: A concept analysis. International Journal of Nursing Studies, 99, 1–8. 10.1016/j.ijnurstu.2019.103386 31404821

[bibr54-10436596241301407] Shopo K. Rabie T. Du Preez A. Bester P. (2023). Experiences of midwives regarding provision of culturally competent care to women receiving maternal care in South Africa. Midwifery, 116, 1–7. 10.1016/j.midw.2022.103527 36323078

[bibr55-10436596241301407] Stubbe D. E. (2020). Practicing cultural competence and cultural humility in the care of diverse patients. Focus, 18(1), 49–51. 10.1176/appi.focus.20190041 32047398 PMC7011228

[bibr56-10436596241301407] Toracco R. J. (2005). Writing integrative literature reviews: Guidelines and examples. Human Resource Development Review, 4(3), 356–367.

[bibr57-10436596241301407] Tosun B. Yava A. Dirgar E. Şahin E. B. Yılmaz E. B. Papp K. Tóthova V. Hellerova V. Prosen M. Licen S. Karnjus I. Tamayo M. D. B. Leyva-Moral J. M. Claeys A. Tricas-Sauras S. (2021). Addressing the effects of transcultural nursing education on nursing students’ cultural competence: A systematic review. Nurse Education in Practice, 55, 1–10. 10.1016/j.nepr.2021.103171 34388616

[bibr58-10436596241301407] Ulvund I. Mordal E. (2017). The impact of short-term clinical placement in a developing country on nursing students: A qualitative descriptive study. Nurse Education Today, 55, 96–100. 10.1016/j.nedt.2017.05.013 28570945

[bibr59-10436596241301407] Verkuyl M. Atack L. (2024). Ten tips for successful virtual simulation integration in the curriculum. Clinical Simulation in Nursing, 88, 1–4. 10.1016/j.ecns.2024.101516

[bibr60-10436596241301407] Wall-Bassett E. D. Hegde A. V. Craft K. Orbelin A. L. (2018). Using Campinha-Bacote’s framework to examine cultural competence from an interdisciplinary international service-learning program. Journal of International Students, 8(1), 274–283. http://jistudents.org/doi:10.5281/zenodo.1134303

[bibr61-10436596241301407] Zarzycka D. Chrzan-Rodak A. Bąk J. Niedorys-Karczmarczyk B. Ślusarska B. (2020). Nurse Cultural Competence-cultural adaptation and validation of the Polish version of the Nurse Cultural Competence Scale and preliminary research results. PLOS ONE, 15, 1–21. 10.1371/journal.pone.0240884 PMC7567385 33064767

